# Indication and Usefulness of Bile Juice Cytology for Diagnosis of Gallbladder Cancer

**DOI:** 10.1155/2018/5410349

**Published:** 2018-04-18

**Authors:** Hiroshi Itsuki, Masahiro Serikawa, Tamito Sasaki, Yasutaka Ishii, Ken Tsushima, Yoshinari Furukawa, Yoshiaki Murakami, Koji Arihiro, Kazuaki Chayama

**Affiliations:** ^1^Department of Gastroenterology and Metabolism, Graduate School of Biomedical & Health Sciences, Hiroshima University, Hiroshima, Japan; ^2^Department of Gastroenterology, Hiroshima Prefectural Hospital, Hiroshima, Japan; ^3^Department of Gastroenterology, Hiroshima Red Cross Hospital & Atomic-bomb Survivors, Hiroshima Red Cross Hospital & Atomic-bomb Survivors Hospital, Hiroshima, Japan; ^4^Department of Surgery, Applied Life Sciences Institute of Biomedical and Health Sciences, Hiroshima University, Hiroshima, Japan; ^5^Department of Anatomical Pathology, Hiroshima University Hospital, Hiroshima, Japan

## Abstract

**Aim:**

We examined the effectiveness of bile juice cytology for distinguishing between benign and malignant gallbladder lesions of the protruding type with various sampling points, sampling methods, and macroscopic forms in order to discuss the effectiveness of the endoscopic transpapillary gallbladder drainage (ETGD) cytology.

**Methods:**

We studied 162 cases of patients with a lesion localized within the gallbladder. At first, we examined the effectiveness for diagnosis of ETBD cytology using ERC and then that of the first ETGD cytology after placing the ETGD. Next, we examined the diagnostic effectiveness of the washed ETGD cytology by using the ETGD. Finally, we examined complications.

**Results:**

In the final diagnoses, we identified 33 cases of adenocarcinoma, 10 cases of adenoma, 63 cases of ADM, 35 cases of nonneoplastic polyp, and 21 cases of chronic cholecystitis. It was found that the sensitivity of ETBD cytology was 3.6% and that of ETGD cytology was 59.1%. In the comparison of diagnostic effectiveness of cytologic diagnosis using samples of bile juice from the gallbladder collected by different methods, the sensitivities were 38.9% and 73.3% for the first and washed ETGD cytologies, respectively. In the comparison of the diagnostic effectiveness of gallbladder bile juice cytology using samples collected for different forms of lesion and by different methods, the sensitivities were 38.9% and 73.3%, respectively, for the first and washed ETGD cytologies for flat gallbladder wall thickening, while it was impossible to diagnose for lesions of GB polyp.

**Conclusion:**

For diagnosis of gallbladder cancer, we consider that the ETGD cytology should be taken into consideration for lesions of flat gallbladder wall thickening, for which it is difficult to distinguish between benign and malignant lesions.

## 1. Introduction

**Figure 1:**
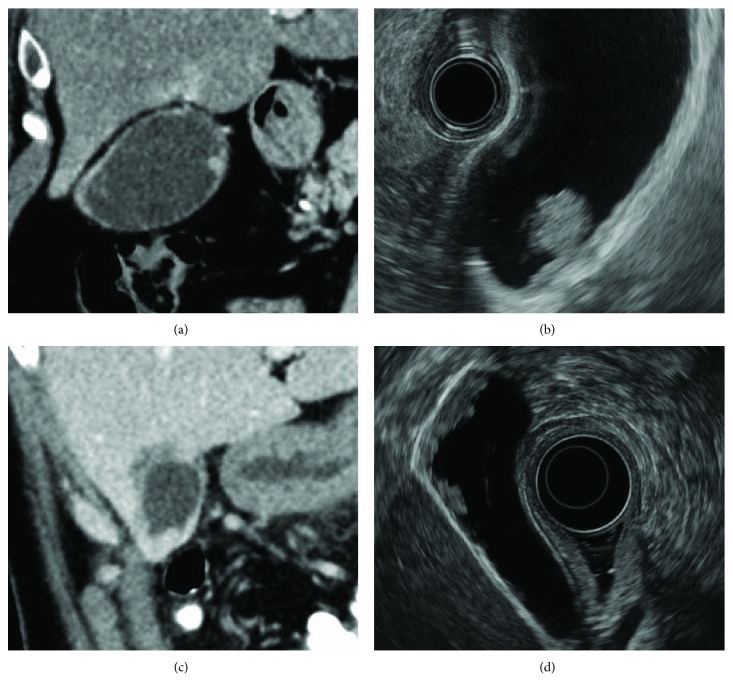
Forms of protruding type of gallbladder lesion. (a, b) A protrusion that exhibits a contrast effect is found in the gallbladder body in an abdominal CT scan image. It abruptly rises to show a pedunculated form. Classified to be a GB polyp based on the EUS image. The site was at the bottom of the gallbladder and the invasion depth was Tis, and it was a case of T1N0M0 Stage I. (c, d) Localized wall thickening that exhibits a contrast effect is found at the bottom of the gallbladder in an abdominal CT scan image. Classified to be a flat gallbladder wall thickening based on the EUS image. The site was at the bottom of the gallbladder and the invasion depth was ss, and it was a case of T2N1M0 Stage IIIb.

Recent improvements in diagnostic imaging technology have revealed characteristics of gallbladder cancer in images [1, 2]. Also, diagnosis methods are now being organized systematically. However, there are still many clinical cases that are difficult to diagnose [3, 4]. Gallbladder lesions of the protruding type are roughly classified based on their macroscopic forms into gallbladder polyps (GB polyps) and flat gallbladder wall thickenings. The cholesterol polyp is the type of GB polyp that is found most frequently. However, gallbladder adenomas and adenoma cancers are also found as GB polyps (Figures 1(a) and 1(b)). Some flat gallbladder wall thickenings are represented in images as sessile polyps as well as wall thickenings. They include early-stage cancer and advanced cancer that has advanced to the SS stage or further, in addition to nonneoplastic lesions of the adenomyomatosis (ADM) and xanthogranulomatous cholecystitis (XGC) (Figures 1(c) and 1(d)). Because the surgical resection rate for gallbladder cancers is not exactly high, and considering the prognosis, it is required to diagnose it in its early stages [5–7]. Pathological diagnosis using bile juice cytology is an important method because it plays a significant role in definitive differential diagnosis between benign and malignant tumors and determination of the treatment method. In particular, it was recently reported that ETGD cytology using bile juice collected by endoscopic transpapillary gallbladder drainage (ETGD) is useful for diagnosis [3, 4]. However, there are still a large number of unresolved issues that must be resolved regarding its application and interpretation.

In this study, we examined the diagnostic effectiveness of bile juice cytology for differential diagnosis between benign and malignant gallbladder lesions of the protruding type with various sampling points, sampling methods, and macroscopic forms in order to discuss the application and effectiveness of the ETGD cytology.

## 2. Patients and Method

### 2.1. Patients and Protocol

We studied 162 cases of patients who were examined in our clinics and whose lesions were localized within the gallbladder. Among these cases, surgical resection and histopathological diagnosis were conducted in 102 cases and follow-up observation was conducted in the remaining 60 cases. For all the cases, the lesions were classified based on their forms, as identified in endoscopic ultrasonography (EUS), and then bile juice was collected by a transpapillary method under ERC.

### 2.2. Procedure for Sampling Bile

**Figure 2:**
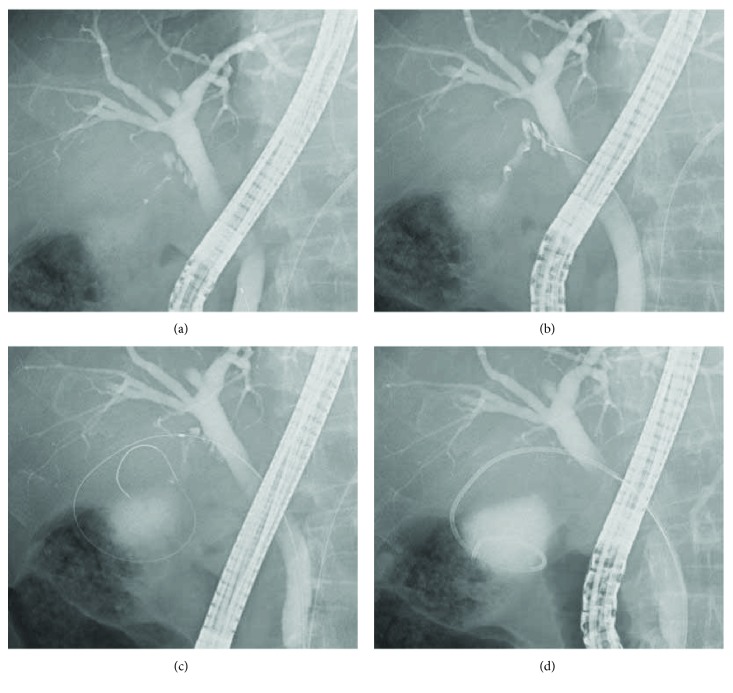
ETGD placement method. (a) Contrast is increased to enable location of the branching point between the bile duct and the cystic duct. (b) The tip of the guiding wire is advanced into the cystic duct. (c) The guiding wire is turned at least one rotation in the gallbladder. (d) A pernasal bile drainage tube of 5 Fr pigtail type is put in place.

Bile juice from the bile duct and bile juice from the gallbladder were collected as the sample for cytologic examination under endoscopic retrograde cholangiography (ERC). For the bile juice from the bile duct, a sufficient amount was siphoned through the imaging catheter after deep intubation into the bile duct. For the bile juice from the gallbladder, a guiding wire of 0.025-inch diameter with an angled tip was inserted into the gallbladder under ERC while carefully seeking the inside of the cystic duct. The guiding wire was turned at least one rotation, and a pernasal bile drainage tube of 5 Fr pigtail type (Flexima Nasobiliary Catheter, Boston Scientific Corporation) was placed in the gallbladder (Figures 2). The bile juice from the gallbladder was first siphoned and collected for cytologic examination just after the ETGD was placed (first ETGD cytology). The next day, the inside of the gallbladder was washed with normal saline solution using the ETGD, and the collected sample of wash solution was used for the cytologic examination (washed ETGD cytology). When a lot of debris was found in the sample of wash solution, the inside of the gallbladder was further washed sufficiently to siphon off and remove the debris.

## 3. Results

### 3.1. Classification of Cases

In the final diagnoses, we identified 33 cases of adenocarcinoma, 10 cases of adenoma, 63 cases of ADM, 35 cases of nonneoplastic polyp, and 21 cases of chronic cholecystitis. In terms of the classification of form, 43 and 119 cases were classified as GB polyp and flat gallbladder wall thickening, respectively. The cases of GB polyp consisted of 5 cases of adenocarcinoma, 10 cases of adenoma, and 28 cases of nonneoplastic polyp. All the adenocarcinomata were carcinomas in adenoma. The cases of flat gallbladder wall thickening consisted of 28 cases of adenocarcinoma, 63 cases of ADM, 7 cases of nonneoplastic lesion, and 21 cases of chronic cholecystitis (Table 1).

### 3.2. Comparison of Diagnostic Effectiveness for Sampling Point of Bile Juice

At first, we examined the diagnostic effectiveness of bile juice cytology with various sampling points. For the diagnostic effectiveness of ETBD cytology, we found 3.6% sensitivity, 100% specificity, 80% accuracy, 100% positive predictive value (PPV), and 80.2% negative predictive value (NPV). Next, we examined the diagnostic effectiveness of ETGD cytology. For the diagnostic effectiveness of ETGD cytology, we found 59.1% sensitivity, 100% specificity, 93.2% accuracy, 100% PPV, and 92.5% NPV. As such, the results for ETGD cytology were better than those for ETBD cytology (Table 2).

### 3.3. Comparison of Diagnostic Effectiveness for Sampling Method for Bile Juice from the Gallbladder

Next, we compared the diagnostic effectiveness of cytologic diagnosis using samples of bile juice from the gallbladder collected by different methods. For the first ETGD cytology using samples collected just after the ETGD was placed, we found 38.9% sensitivity, 100% specificity, 88.7% accuracy, 100% PPV, and 87.8% NPV. On the other hand, for the washed ETGD cytology using samples collected one day after the ETGD was placed, we found 73.3% sensitivity, 100% specificity, 95.4% accuracy, 100% PPV, and 94.8% NPV. The sensitivity and accuracy were improved in the washed ETGD cytology (Table 3).

### 3.4. Comparison of Diagnosability of Lesion Form in the First and Washed ETGD Cytologies

Finally, we examined the diagnostic effectiveness of the first and washed ETGD cytologies for different forms of the lesion. In the examination of bile juice from the gallbladder for GB polyp, no case was diagnosed as malignant for either the first or the washed ETGD cytology (data not shown). On the other hand, for flat gallbladder wall thickening, the ETGD cytology indicated 50% sensitivity, 100% specificity, 88.2% accuracy, 100% PPV, and 86.7% NPV. The sensitivity varied based on the form of lesion (Table 4).

### 3.5. Accidental Symptoms Caused by ETGD Placement

Among the 162 cases we examined in this study, we identified an accidental symptom in 14 cases in total (8.6%). These consisted of 1 case of obstructive jaundice, 3 cases of acute cholecystitis, 9 cases of acute pancreatitis, and 1 case of gallbladder perforation.

## 4. Discussion

Diagnosability of gallbladder cancer has been improved recently thanks to the improvements in ultrasonography (US) and EUS and their use in combination with multidetector-row computed tomography (MDCT) and magnetic resonance imaging (MRI) systems [8, 9]. Although findings from the examination of these images are of great importance in the diagnosis of gallbladder cancers, they include cases in which it is difficult to distinguish gallbladder cancers such as XGC and ADM, and unnecessarily large-scale operations are in some cases conducted [3, 10, 11]. Therefore, pathological diagnosis using bile juice cytology is an important method because it plays a significant role in the definitive differential diagnosis between benign and malignant tumors and the determination of treatment method.

The ETGD placement method was first conducted by Kozarek [12] in 1984, and it is said to be effective and safe for cases in which percutaneous drainage is difficult to conduct due to high risk of bleeding or acute cholecystitis with ascites [13]. Itoi et al. reported that the success rate of ETGD placement for gallbladder diseases was 81%. For 162 cases in which we tried to perform selective intubation into the gallbladder, the success rate of selective insertion of the guiding wire was 80.9%, which indicates almost the same level. It was possible to place the drainage tube in all the cases in which the guiding wire was successfully inserted to the gallbladder. Itoi et al. pointed out some factors for successful ETGD placement for gallbladder diseases, such as accurate determination of the location of the branching point between the bile duct and the cystic duct, coordinated handling of the cannula and the guiding wire for the spirally winding cystic duct, and use of a swing catheter tip for cases in which the cystic duct branches downward from the bile duct [14]. Many examinations have been conducted for the treatment of acute pancreatitis using the ETGD that is placed in the gallbladder [15]. On the other hand, only a few studies have been reported for diagnosis of gallbladder cancer based on ETGD cytology using the ETGD placed in the gallbladder to collect the sample.

In this study, we examined the effectiveness of bile juice cytology for differential diagnosis between benign and malignant gallbladder lesions of the protruding type. In the comparison of diagnostic effectiveness of various sampling points for bile juice, we found that the sensitivity of ETGD cytology increased to 59.1% from 3.6% for ETBD cytology. Naito et al. also reported that ETGD cytology showed a superior sensitivity of 78% for gallbladder cancer compared to 20% sensitivity for ETBD cytology [16]. Based on these results, it was concluded that the sample should be collected at a point near the lesion, inside the gallbladder, for differential diagnosis between benign and malignant gallbladder tumors.

Then we examined the sensibility of ETGD cytology for different macroscopic forms of lesion. The sensitivity for flat gallbladder wall thickening was 50%, while the sensitivity for GB polyp was 0%. In addition to that, we compared diagnostic effectiveness of sampling methods for bile juice from the gallbladder for flat gallbladder wall thickening. While the sensitivity in the first ETGD cytology for flat gallbladder wall thickening was 38.9%, the sensitivity of the washed ETGD cytology was improved to 73.3%. Tamada et al. also reported that the diagnostic effectiveness of cytologic diagnosis for gallbladder cancer using the first ETGD cytology was low and was improved by using the washed ETGD cytology while the ETGD was in place [15]. It is assumed that this is because the sample collected for the first ETGD cytology contains a lot of inflammatory cells and biliary sludge, and the epithelial cells of the gallbladder are altered. In the washed ETGD cytology, on the other hand, fresh exfoliated cells are collected due to the washing process. Matsubayashi et al. [17] reported that the washed ETGD cytology using ETGD was very effective for a case in which a malignant black tumor had spread into the stroma of the mucosa of the gallbladder. In this study, it was found that the cytologic diagnosis method is not capable of identifying gallbladder cancer of the GB polyp type, regardless of whether the sample is collected in the bile duct or the gallbladder. It was concluded that this was because all the malignant lesions of the GB polyp type were adenocarcinomata, and the volume of the tumor that constitutes the malignant lesion was rather small.

Considering the results described above, it was concluded that, considering the risk of complications, ERC is not required in the case of a GB polyp which is suspected to be a malignant lesion based on the image, and laparoscopic cholecystectomy should be performed immediately. However, needless to say, it is important to observe the lesion in detail using EUS since some lesions concurrently contain GB polyp and flat gallbladder wall thickening. On the other hand, it is thought that the effectiveness of the ETGD cytology for diagnosis of gallbladder cancer in flat gallbladder wall thickening is very high, particularly for the washed ETGD cytology using ETGD. Therefore, for diagnosis of flat gallbladder wall thickening, which is suspected to be gallbladder cancer, it is required to consider differential diagnosis between benign and malignant lesions using the ETGD cytology, in addition to various imaging diagnostic methods, in order to carefully determine the surgical procedure to be adopted.

When placing the ETGD, it is required to take the utmost care to avoid complications. Itoi et al. reported that they experienced a complication in 8.2% of 330 cases in which the ETGD was placed. They reported 2 cases of gallbladder perforation, 2 cases of bile leak, 1 case of bleeding, and 22 cases of the other complications. Among the 162 cases we examined in this study, we identified an accidental symptom in 14 cases in total (8.6%). These consisted of 1 case of obstructive jaundice, 3 cases of acute cholecystitis, 9 cases of acute pancreatitis, and 1 case of gallbladder perforation. However, we did not experience any fatal complication, and all patients recovered after removal of the tube and/or conservative treatment. For the placement of ETGD, Mori et al. described that selective cannulation into the bile duct and the appropriate handling of the guiding wire are required, because a serious complication, such as pancreatitis or gallbladder perforation, can be caused after the ERCP [18].

We have described the results of our study on the effectiveness of bile juice cytology for differential diagnosis between benign and malignant gallbladder lesions of the protruding type. Needless to say, imaging diagnosis methods take a central role in the diagnosis of gallbladder cancer. However, we take the view that the ETGD cytology should be positively taken into consideration for lesions of flat gallbladder wall thickening for which it is difficult to distinguish between benign and malignant lesions and to determine the treatment policy, particularly the surgical procedure, to be adopted.

## Figures and Tables

**Table 1:** Details of protruding type of gallbladder lesion.

	GB polyp	Flat gallbladder wall thickening
Adenocarcinoma	5	28
Adenoma	10	0
Adenomyosis	0	63
Nonneoplastic polyp	28	7
Chronic cholecystitis	0	21
Total	43	119

**Table 2:** Diagnostic effectiveness comparison for ETBD cytology and ETGD cytology.

	Bile duct bile juice (*n* = 137)	Gallbladder bile juice (*n* = 133)
Sensitivity	3.6%	59.1%
Specificity	100%	100%
Accuracy	80.1%	93.2%
PPV	100%	100%
NPV	80.2%	92.5%

**Table 3:** Diagnostic effectiveness comparison for first and washed ETGD cytologies.

	First ETGD cytology (*n* = 97)	Washed ETGD cytology (*n* = 88)
Sensitivity	38.9%	73.3%
Specificity	100%	100%
Accuracy	88.7%	95.4%
PPV	100%	100%
NPV	87.8%	94.8%

**Table 4:** Diagnostic effectiveness of ETGD cytology for forms of lesion.

	GB polyp (*n* = 43)	Flat gallbladder wall thickening (*n* = 119)
Sensitivity	0.0%	50.0%
Specificity	100%	100%
Accuracy	88.4%	88.2%
PPV	0.0%	100%
NPV	88.4%	86.7%
